# Immunohistochemistry for (Pro)renin Receptor in Humans

**DOI:** 10.1155/2021/8828610

**Published:** 2021-07-27

**Authors:** Satoshi Morimoto, Noriko Morishima, Daisuke Watanabe, Yoichiro Kato, Noriyuki Shibata, Atsuhiro Ichihara

**Affiliations:** ^1^Department of Endocrinology and Hypertension, Tokyo Women's Medical University, Tokyo, Japan; ^2^Department of Pathology, Tokyo Women's Medical University, Tokyo, Japan

## Abstract

The (pro)renin receptor is a multifunctional protein with roles in angiotensin-II-dependent and -independent intracellular cell signaling and roles as an intracellular accessory protein for the vacuolar H^+^-ATPase, including hormone secretion. While (pro)renin receptor mRNA is widely expressed in various human tissues, localization of (pro)renin receptor protein expression has not yet been systemically determined. Therefore, this study localized (pro)renin receptor protein expression in human organs. Systemic immunohistochemical examination of (pro)renin receptor expression was performed in whole body organs of autopsy cases. (Pro)renin receptor immunostaining was observed in the cytoplasm of cells in almost all human organs. It was observed in thyroid follicular epithelial cells, hepatic cells, pancreatic duct epithelial cells, zona glomerulosa and zona reticularis of the cortex and medulla of the adrenal gland, proximal and distal tubules and collecting ducts of the kidney, cardiomyocytes, and skeletal muscle cells. In the brain, (pro)renin receptor staining was detected in neurons throughout all areas, especially in the medulla oblongata, paraventricular nucleus and supraoptic nucleus of the hypothalamus, cerebrum, granular layer of the hippocampus, Purkinje cell layer of the cerebellum, and the pituitary anterior and posterior lobes. In the anterior lobe of the pituitary gland, all types of anterior pituitary hormone-positive cells showed double staining with (pro)renin receptor. These data showed that (pro)renin receptor protein was expressed in almost all organs of the human body. Its expression pattern was not uniform, and cell-specific expression pattern was observed, supporting the notion that (pro)renin receptor plays numerous physiological roles in each human organ.

## 1. Introduction

The (pro)renin receptor ((P)RR) consisting of 350 amino acids with a single transmembrane domain binds preferentially to renin and prorenin [1]. The binding of prorenin to the (P)RR leads to nonproteolytic renin activation [2], which induces the conversion of angiotensinogen to angiotensin (Ang) I. This process plays a key role in the regulation of the tissue renin-angiotensin system (RAS) [1]. The (P)RR also stimulates its own intracellular tyrosine-phosphorylation-dependent pathways, such as mitogen-activated protein kinase (MAPK) [1] and extracellular-signal-regulated kinase 1 and 2 (ERK1 and ERK2) [3], independent of RAS activation.

The (P)RR was identified as an accessory protein of vacuolar H^+^-ATPase (V-ATPase), which is an ATP-dependent proton pump that plays important roles in the transportation of protons across plasma membranes and acidifies intracellular compartments [4]. V-ATPase also has a function in secreting hormones and membrane fusion, independent of acidification [5–7]. The (P)RR also works as an adaptor protein between the V-ATPase and Wnt receptor complex [8], which is involved in virtually every aspect of embryonic development and in homeostatic self-renewal [9]. In addition, binding of the (P)RR to the pyruvate dehydrogenase E1 *β*-subunit (PDHB) prevents PDHB phosphorylation and maintains aerobic glucose metabolism [10]. Therefore, the (P)RR is a multifunctional protein that exhibits a complex structure and functionality [11].

(P)RR mRNA expression has been detected in various human organs: very high levels in the brain, heart, and placenta, high levels in the liver, kidney, and pancreas, and weak levels in lung and skeletal muscle [1]. However, actual sites of (P)RR protein expression in the whole body remain to be determined. Therefore, this study investigated the localization of (P)RR protein expression in human organs using systemic immunohistochemical examination.

In addition, it has been reported that (P)RR has a functional role in secretion of growth hormone (GH) via V-ATPase from pituitary cells [12], raising the possibility that (P)RR may have roles in secretion of any pituitary hormones. Accordingly, this study also examined whether (P)RR is colocalized with all anterior pituitary hormones.

## 2. Materials and Methods

### 2.1. Subject

The study was performed in accordance with the principles of the Declaration of Helsinki and its amendments and approved by the Ethics Committee of Tokyo Women's Medical University (approval #: 3936). Immunohistochemical experiments were performed using 20% formalin-fixed and paraffin-embedded materials achieved at necropsy less than 16 h after death and after written informed consent being obtained from the family. The expression of (P)RR in whole body organs was obtained from three subjects (subject 1 was a female who had myelodysplastic syndrome and sick sinus syndrome and died of multiple organ damage and congestive heart failure at the age of 65; subject 2 was a female who had myocardial infarction and died of ventricular fibrillation at the age of 82; and subject 3 was a male who had myocardial infarction and died of congestive heart failure at the age of 70) and in the anterior lobe of the pituitary from one male subject who had malignant lymphoma and died of respiratory failure at the age of 77.

### 2.2. Histology and Immunohistochemistry

Multiple sections with 6 and 3 *μ*m thickness were prepared for immunohistochemical staining of the pituitary gland and other tissues, respectively. The sections were deparaffinized, rehydrated, and rinsed in phosphate-buffered saline (PBS) pH 7.6. They were quenched in 3% H_2_O_2_ at room temperature (RT) for 10 min to stop endogenous peroxidase, rinsed in PBS, and treated with 5% skim milk/PBS at RT for 5 min to inhibit binding of nonspecific antibody. They were then incubated at 4°C overnight with primary antibodies against (P)RR (dilution 1 : 200), which were raised in a rabbit by injecting the human (P)RR peptide fragment corresponding to 222–239 amino acids and provided by Immuno-Biological Laboratories Co., Ltd. (Fujioka City, Japan) with or without the human (P)RR peptide fragment corresponding to 222–239 amino acids provided by Immuno-Biological Laboratories Co., Ltd. (Fujioka City, Japan). (P)RR immunoreactivity was detected by the polymer-immunocomplex (PIC) method with the use of the Envision system (Dako, Santa Clara, CA, USA). 3,3′-Diaminobenzidine tetrahydrocholoride (DAB, Dojindo Laboratories, Kumamoto City, Japan) was the chromogen, and hematoxylin was the counterstain. Sections incubated with nonimmune animal serum or processed without a primary antibody were utilized as negative controls. As shown in Table 1, immunohistochemical localization was confirmed by comparing to consecutive sections with hematoxylin and eosin (HE) stainings. In addition, identical sections were double-immunostained for anterior pituitary hormones. The double immunostaining was performed as follows: (1) sections were incubated with (P)RR antibody; (2) binding of antibody was visualized with DAB by the PIC method; (3) immunohistochemical images were microphotographed; (4) sections were rinsed in PBS to preserve DAB pigments; (5) sections were processed with 1% hydrochloric acid 70% alcohol for removal of the GH antibody or adrenocorticotropic hormone (ACTH) antibody, secondary antibody, and hematoxylin and processed by microwave treatment to remove luteinizing hormone (LH) antibody, follicle-stimulating hormone (FSH) antibody, thyroid-stimulating hormone (TSH) antibody, or prolactin (PRL) antibody, secondary antibody, and hematoxylin; (6) sections were incubated with anterior pituitary hormone antibodies; (7) binding of antibody was detected using DAB-NiCl_2_ by the PIC method after DAB; and (8) immunohistochemical observations were compared with images at the same positions as those in the initial pictures. The immunostaining intensity in the cytoplasm was evaluated on a scale of − (no staining), ± (borderline staining), and + (positive staining).

## 3. Results

**Figure 1:**
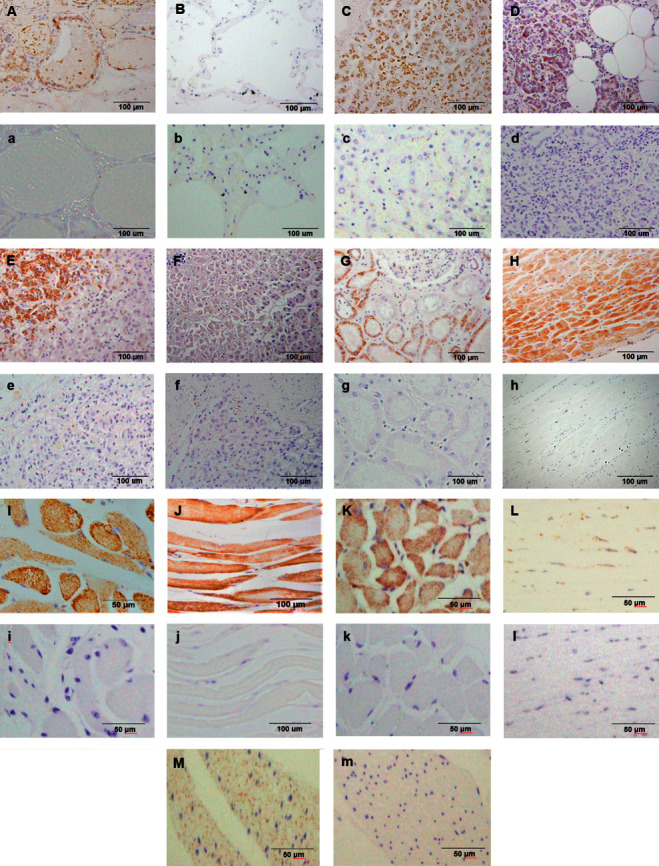
(Pro)renin receptor staining in organs outside the brain. (A) Thyroid gland; (B) lung; (C) liver; (D) pancreas; and (E) cortex of the adrenal gland. Cells in the zona glomerulosa are stained; (F) medulla of the adrenal gland; (G) kidney; (H) left ventricle; (I) lingual muscle; (J) diaphragm; (K) iliopsoas muscle; (L), aorta; and (M) colon. (a–m) Negative controls in the organs mentioned.

(P)RR immunostaining was observed in the cytoplasm of cells in almost all human organs. The distribution of (P)RR expression in organs outside the brain is summarized in Table 2. (P)RR immunostaining was observed in follicular epithelial cells in the thyroid gland (Figure 1(A)), but not in the lungs (Figure 1(B)). (P)RR expression was seen in hepatic cells (Figure 1(C)) and pancreatic duct epithelial cells, but not in the islets of Langerhans cells in the pancreas (Figure 1(D)). In the adrenal glands, (P)RR immunostaining was observed in cells in the zona glomerulosa and zona reticularis of the cortex (Figure 1(E)) and sparsely in cells of the medulla (Figure 1(F)). In the kidneys, proximal and distal tubules and collecting ducts, but not glomeruli, were immunostained (Figure 1(G)). Cardiomyocytes (Figure 1(H)) and skeletal muscle cells (Figures 1(I)–1(K)) were clearly stained, and smooth muscle cells of the aorta (Figure 1(I)) and colon (Figure 1(M)) were weakly stained.

**Figure 2:**
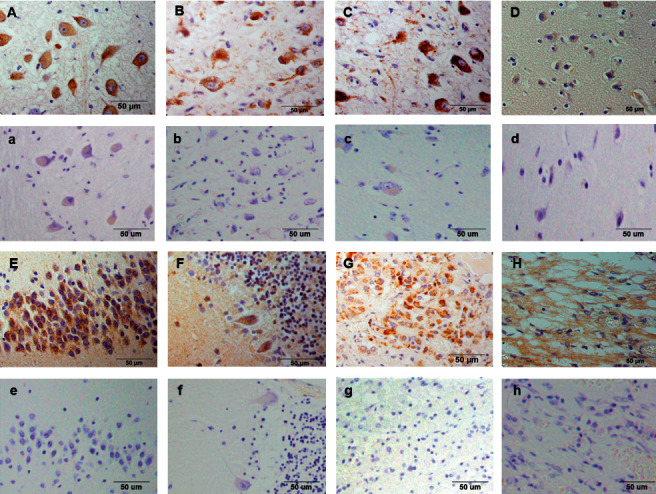
(Pro)renin receptor staining in brain lesions. (A) Nucleus tractus solitarii; (B) paraventricular nucleus of the hypothalamus; (C) supraoptic nucleus of the hypothalamus; (D) cerebrum; (E) granular layer of the hippocampus; (F) Purkinje cell layer of the cerebellum; (G) anterior lobe of the pituitary gland; and (H) posterior lobe of the pituitary gland. (a–h) Negative controls in the organs mentioned.

**Figure 3:**
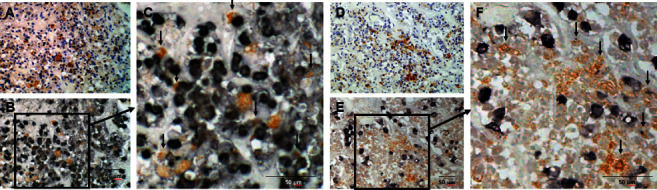
Double staining of (pro)renin receptor and growth hormone or adrenocorticotropic hormone. (A), (D) (Pro)renin receptor staining counterstained with hematoxylin; (B) double staining of (pro)renin receptor and growth hormone in the identical section as (A); (C) high-power field of (B); (E) double staining of (pro)renin receptor and adrenocorticotropic hormone in the identical section as (D); and (F) high-power field of (E). Arrows indicate cells costained with (pro)renin receptor.

**Figure 4:**
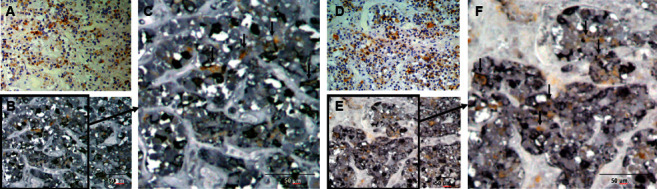
Double staining of (pro)renin receptor and luteinizing hormone or follicle-stimulating hormone. (A), (D) (Pro)renin receptor staining counterstained with hematoxylin; (B) double staining of (pro)renin receptor and luteinizing hormone in the identical section as (A); (C) high-power field of (B); (E) double staining of (pro)renin receptor and follicle-stimulating hormone in the identical section as (D); and (F) high-power field of (E). Arrows indicate cells costained with (pro)renin receptor.

**Figure 5:**
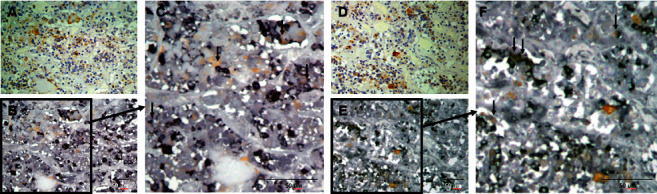
Double staining of (pro)renin receptor staining and thyroid-stimulating hormone or prolactin. (A), (D) (Pro)renin receptor staining counterstained with hematoxylin; (B) double staining of (pro)renin receptor and thyroid-stimulating hormone in the identical section as (A); (C) high-power field of (B); (E) double staining of (pro)renin receptor and prolactin in the identical section as (D); and (F) high-power field of (E). Arrows indicate cells costained with (pro)renin receptor.

**Figure 6:**
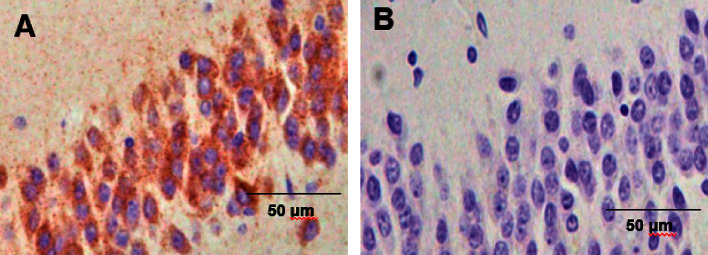
(Pro)renin receptor staining in the granular layer of the hippocampus with (B) or without (A) the human (pro)renin receptor fragment as a blocking peptide. The specificity of the (P)RR antibody used was confirmed.

The distribution of (P)RR expression in the brain is summarized in Table 3. In the brain, (P)RR staining was detected in neurons throughout the brain, especially in the medulla oblongata (Figure 2(A)), paraventricular nucleus (PVN) (Figure 2(B)), and supraoptic nucleus (SON) (Figure 2(C)) of the hypothalamus. (P)RR expression was also observed in the cerebrum (Figure 2(D)), granular layer of the hippocampus (Figure 2(E)), Purkinje cell layer of the cerebellum (Figure 2(F)), and anterior (Figure 2(G)) and posterior lobes (Figure 2(H)) of the pituitary. Double immunostaining of (P)RR and anterior pituitary hormones were performed on the anterior lobe of the pituitary gland. All types of anterior pituitary hormone-positive cells showed double staining with (P)RR, although some pituitary hormone-positive cells were not costained with (P)RR (Figures 3–5). The specificity of the (P)RR antibody used was confirmed by the lack of staining with a (P)RR peptide as a blocking peptide (Figure 6).

## 4. Discussion

In this study, (P)RR immunostaining was observed in the cytoplasm of cells in almost all human organs. However, the expression pattern of (P)RR was not uniform and cell-specific expression was observed in these organs. Because (P)RR is an accessory protein of V-ATPase, which is essential for cell survival and is expressed in all cells [4], (P)RR should be expressed in all cells. Therefore, it could be possible that the expression levels of (P)RR associated with V-ATPase function may be very low and cells expressing (P)RR associated with other functions may be detected as (P)RR-positive cells.

### 4.1. (P)RR Expression in the Liver

(P)RR mRNA is expressed in the liver [1], and this study showed (P)RR protein is expressed in human hepatocytes (Figure 1(c)). The liver has important roles in the regulation of low-density lipoprotein (LDL). Sortilin-1 (SORT1) is a regulator of LDL metabolism [13–15]. Overexpression of SORT1 increases LDL clearance and decreases plasma LDL levels [14–16], whereas SORT1 deficiency reduces cellular LDL uptake *in vitro* and LDL clearance *in vivo* [15, 17]. The (P)RR interacts with SORT1 [18]; silencing of (P)RR expression in hepatocytes *in vitro* reduces protein abundance of SORT1 and low-density lipoprotein receptor posttranscriptionally and, consequently, cellular LDL uptake [19]. In addition, (P)RR plays a key role in energy homeostasis and regulation of plasma lipids by integrating hepatic glucose and lipid metabolism [19]. Therefore, the role of (P)RR in the liver is an interesting area for further investigation.

### 4.2. (P)RR Expression in the Heart

(P)RR mRNA expression levels are extremely high in the human heart [1]. In the present study, (P)RR protein expression was detected in human cardiomyocytes (Figure 1(h)). A previous report indicated that gene expression of (P)RR, renin, and angiotensinogen is increased in the heart with chronic heart failure in rats [20]. High salt intake enhances the cardiac expression of (P)RR, causing acceleration of interstitial fibrosis, perivascular fibrosis, and hypertrophy in the heart via Ang-II-dependent and -independent pathways at an early stage of hypertension [21]. Overexpression of (P)RR in the heart shows deleterious effects on cardiac function via activating Ang-II-independent extracellular matrix remodeling in rats [22]. Therefore, (P)RR is expected to have important roles via Ang-II-dependent and/or -independent mechanisms, which should be investigated in future studies.

### 4.3. (P)RR Expression in the Kidneys

(P)RR mRNA expression is detected in the human kidney [1]. In addition, (P)RR immunostaining is detected throughout the nephron, especially in the collecting ducts, distal convoluted tubules, and distal tubules in normal kidney sections of patients who have undergone nephrectomy for renal cell carcinoma [23]. In human kidney tissues obtained at autopsy from patients with end-stage renal disease, (P)RR immunostaining is mainly detected in the tubular cells and collecting duct cells with weak and sporadic staining in the glomeruli [24]. In the present study, (P)RR immunostaining was observed in proximal and distal tubules and collecting ducts, without definite immunostaining in the glomeruli (Figure 1(g)). (P)RR is essential for normal nephron development, possibly via regulation of lysosomal acidification, and is important in regulating blood pressure via renal sodium and water and acid excretion under physiological conditions [25].

### 4.4. (P)RR Expression in the Pancreas

Low levels of (P)RR mRNA expression are observed in the human pancreas [1]. In rats, the (P)RR participates in the pathogenesis of glucose intolerance, at least in part through RAS-dependent mechanisms [26, 27]. In addition, the a3 isoform of the V-ATPase has been shown to be expressed in the islets of Langerhans and to have a pivotal role in the regulation of insulin exocytosis from pancreatic *β*-cells in mice [28]. In the present study, (P)RR expression was observed in pancreatic duct epithelial cells (Figure 1(d)). However, unexpectedly, (P)RR expression was not detected in the islets of Langerhans. It could be possible that basal (normal) expression levels of (P)RR associated with V-ATPase are too low to be detected by immunohistochemistry; however, this presumption is speculative and should be tested by future studies. The role of the (P)RR in the pancreas also requires further investigation.

### 4.5. (P)RR Expression in the Brain

In the brain of mice, the (P)RR protein is expressed mostly in the neurons of the nucleus tractus of solitarii (NTS), rostral ventral lateral medulla (RVLM), PVN, subfornical organ (SFO), and the area postrema (AP) [29], which are considered to be the cardiovascular regulatory regions of the brain. Evidence from rodent models suggest a role of the (P)RR in the central regulation of blood pressure in an RAS-dependent [29, 30] and RAS-independent manner [31]. (P)RR mRNA expression levels are extremely high in the human brain [1]; (P)RR mRNA is widely expressed in the brain, with the highest expression in the pituitary gland and frontal lobe, while the (P)RR protein is expressed in the PVN and SON of the hypothalamus and in the anterior lobe of the pituitary [32]. In the present study, (P)RR immunostaining was detected in neurons throughout the brain, especially in cardiovascular regulatory regions for water-electrolyte metabolism and blood pressure, such as the medulla oblongata (Figure 2(A)), PVN (Figure 2(B)) and SON (Figure 2(C)) of the hypothalamus, and the anterior (Figure 2(G)) and posterior lobes (Figure 2(H)) of the pituitary. (P)RR has a functional role in GH secretion via V-ATPase from pituitary GH-producing tumor cells [12]. In the present study, all anterior pituitary hormones showed costaining with the (P)RR, suggesting that the (P)RR may have functional roles in secretion of these hormones in the normal anterior lobe of the pituitary. These data raised the possibility that the (P)RR plays important physiological roles, not only in the central control of water-electrolyte metabolism and blood pressure but also in the neuroendocrine control of pituitary hormone secretions and other brain functions. It is also a fact that some pituitary-hormone-positive cells were not costained with (P)RR (Figures 3–5). The reason for this is unclear, however, it could be possible that the expression levels of (P)RR in some hormone-positive cells may be too low to be detected by immunohistochemistry.

### 4.6. (P)RR Expression in Other Endocrine Organs

In this study, (P)RR immunostaining was observed in follicular epithelial cells in the thyroid gland (Figure 1(A)). In the normal adrenal gland, positive (P)RR immunostaining is observed in both the adrenal cortex and medulla, with higher (P)RR immunostaining observed in the zona glomerulosa and zona reticularis [33]. In this study, (P)RR immunostaining was observed in cells in the zona glomerulosa, in the zona reticularis of the cortex (Figure 1(E)), and sparsely in cells in the medulla (Figure 1(F)). These data further suggest the possibility that the (P)RR may have functional roles in the secretion of hormones in endocrine organs. Further studies are required to investigate the roles of (P)RR in hormone secretion in more detail.

### 4.7. Limitations

There are some limitations to this study. First, because archival human specimens obtained at autopsy were investigated, the ureter, bladder, urethra, uterus, ovaries, and placenta could not be analyzed. Second, the sample size was small. Third, the functional roles of (P)RR could not be determined by this immunohistochemical study. However, this study demonstrated, for the first time, the regional distribution of (P)RR protein expression systemically in human organs, providing important information on the roles of (P)RR in humans.

## 5. Conclusions

In the present study, (P)RR immunostaining was observed in almost all human organs. The expression pattern of (P)RR was not uniform, and cell-specific expression patterns were observed in these organs. These findings support the notion that the (P)RR plays numerous physiological roles, including hormone secretion, in humans. Further studies are required to determine the functions of the (P)RR in detail in each organ.

## Figures and Tables

**Table 1:** Primary antibodies used in immunohistochemistry of anterior pituitary hormones.

Antigen	Animal/clonality	Cat. no./clone	Dilution	Pretreatment	Source
GH	Mouse/mAb	sc-374266	1 : 100	NN	Santa Cruz Biotechnology
ACTH	Mouse/mAb	AM32828PU-N	1 : 200	NN	Acris Antibodies
LH	Mouse/mAb	MS-9078-P1	1 : 1000	MW	Lab Vision
FSH	Mouse/mAb	MS-1449-P1	1 : 500	MW	Lab Vision
TSH	Mouse/mAb	MS-1453-P1	1 : 200	MW	Lab Vision
PRL	Mouse/mAb	MS-9083-P1	1 : 200	MW	Lab Vision

GH, growth hormone; ACTH, adrenocorticotropic hormone; LH, luteinizing hormone; FSH, follicle-stimulating hormone; TSH, thyroid-stimulating hormone; PRL, prolactin; mAb, monoclonal antibody; MW, microwaving in citrate buffer, pH 6.0 (15 min, 95°C, 400 W); NN, not necessary.

**Table 2:** Distribution of (pro)renin receptor expression in organs outside the brain.

	Case 1	Case 2	Case 3
*Thyroid*			
Follicular epithelial cell	+	−	+
Lymph duct	−	−	−

*Lung*			
Alveoli epithelial cell	−	−	−
Blood vessel	−	−	−

*Liver*			
Hepatic cell	+	+	+
Epithelial cell	−	−	−
Cholangioepithelial cell	−	−	−
Blood vessel	−	−	−
Lymph duct	−	−	−

*Pancreas*			
Acinar cell	−	−	−
Pancreatic duct epithelial cell	−	−	+
Islets of Langerhans	−	−	−
Blood vessel	−	−	−
Lymph duct	−	−	−

*Adrenal gland*			
Zona glomerulosa	+	−	+
Zona fasciculate	−	−	−
Zona reticularis	−	−	±
Medulla	+	−	+

*Kidney*			
Glomerulus	−	−	−
Proximal tubule	−	+	+
Distal tubule	±	+	+
Collecting duct	+	+	+
Blood vessel	−	−	−
Lymph duct	−	−	−

*Skeletal muscle*			
Lingual muscle	N/A	−	+
Diaphragm	+	N/A	+
Iliopsoas muscle	N/A	N/A	±
Cardiomyocytes	+	+	+

*Smooth muscle*			
Aorta	N/A	−	+
Colon	±	−	−

The immunostaining intensity in the cytoplasm was evaluated on a scale of − (no staining), ± (borderline staining), and + (positive staining). N/A, not available.

**Table 3:** Distribution of (pro)renin receptor expression in the brain.

	Case 1	Case 2	Case 3
*Brain stem*			
Medulla			
Nucleus tractus solitarii	+	+	+
Dorsal nucleus of vagus	+	+	+
Hypoglossal nucleus	+	+	+
Inferior olivary nucleus	+	+	+
Midbrain			
Thalamus	+	+	+
Lateral nucleus	+	+	+
Medial nucleus	+	+	+
Hypothalamus			
Paraventricular nucleus	+	+	+
Supraoptic nucleus	N/A	+	+
Mammillary body	+	+	+

*Cerebrum*			
Grey matter	+	+	+
White matter	+	N/A	N/A
Hippocampus			
Stratum radiatum	±	+	+
Pyramidal cell layer	+	+	+
Stratum lacunosum	±	+	+

*Cerebellum*			
Molecular layer	±	±	±
Purkinje cell layer	+	+	+
Granular layer	±	±	±

*Pituitary*			
Anterior lobe	+	+	+
Posterior lobe	−	−	+

The immunostaining intensity in the cytoplasm was evaluated on a scale of − (no staining), ± (borderline staining), and + (positive staining). N/A, not available.

## Data Availability

No data were used to support this study.
